# Assessment of Fear of Failure Among Medical Students at King Saud University

**DOI:** 10.3389/fpsyg.2022.794700

**Published:** 2022-03-11

**Authors:** Abeer Alabduljabbar, Lyan Almana, Alanoud Almansour, Aljoharah Alshunaifi, Nada Alobaid, Norah Alothaim, Shaffi Ahamed Shaik

**Affiliations:** ^1^College of Medicine, King Saud University, Riyadh, Saudi Arabia; ^2^SABIC Psychological Health Research and Applications Chair (SPHRAC), Psychiatry Department, College of Medicine, King Saud University, Riyadh, Saudi Arabia; ^3^Department of Family and Community Medicine, College of Medicine, King Saud University, Riyadh, Saudi Arabia

**Keywords:** fear of failure (FoF), fear, medical students, medical education, academic achievements

## Abstract

**Background:**

Fear of failure (FoF) is described as a “dispositional tendency to avoid failure in achievement settings.” It may potentially and adversely affect students’ ability to perform well in their educational activities.

**Objectives:**

To measure FoF among medical students at King Saud University, FoF between men and women, academic levels, grade point average (GPA), and other factors among medical students were compared.

**Method:**

A cross-sectional observational study was carried out using a stratified random sampling method. A total of 455 medical students completed “the Performance Failure Appraisal Inventory” during the academic year 2019–2020 at King Saud University, Riyadh, Saudi Arabia.

**Results:**

The results showed that the mean of FoF was −0.3117. Moreover, higher levels of fear of devaluing one’s self-estimate were seen in women, and higher levels of fear of important others losing interest were seen in men. A significant relation was seen between different academic levels and fear of shame and embarrassment, fear of upsetting important others, as well as FoF. Higher levels of FoF were seen in those who had a GPA below 3.5 and a GPA greater than 4.9. Also, it was high in students who were not interested in studying medicine. The Cronbach’s α value of 0.93 of all items indicates good internal consistency, and the factor analysis confirms five items of an instrument.

**Conclusion:**

The overall level of FoF was low among medical students at King Saud University. However, the domains and levels of FoF differed significantly according to gender, academic level, GPA, and interest in studying medicine.

## Introduction

Fear of failure (FoF) is described as a “dispositional tendency to avoid failure in achievement settings because the humiliation and embarrassment of failure are perceived to be overwhelming” (Conroy and Elliot, 2004). Earlier, it was described as two different motivational behaviors: the first one is identified as the motivation to seek success, and the other one is the desire to escape failure (Elliot and Sheldon, 1997). Avoiding failure pushes the individual to protect the self from the consequences of failure by utilizing cognitive methods such as “self-handicapping” (Elliot and Thrash, 2004; Bartels and Herman, 2011). These self-imposed obstacles such as setting unfeasible goals or procrastinating, not studying for an exam until the last minute, diverting the perceived cause of failure from personal causes such as lack of ability to environmental causes that cannot be controlled by the individual (Covington, 1984; Bartels and Herman, 2011).

It was previously reported in the literature that procrastination in academic tasks has been a source of difficulty for medical students (Hayat et al., 2020). FoF may potentially and adversely affect the ability of students to perform well in their educational activities, yet low levels of FoF may encourage some students to improve their skills and develop new academic performance strategies (Nsiah, 2017). A study done in a university counseling center during one academic year revealed that 35% of university music students have FoF (Conroy, 2001). FoF has numerous detrimental effects on students, especially in those who are failure avoidant. It is associated with higher anxiety levels, self-doubt, and lower self-estimate (Elliot and Thrash, 2004; Martin and Marsh, 2003).

In a study done among dental university students, examinations on a regular basis, academic curriculum immensity, low examination achievement, and FoF were found to be important stimuli for stress (Srivastava et al., 2020). Less satisfaction and enjoyment while completing their task were also reported (Elliot and Sheldon, 1997). Moreover, past studies have shown that FoF affects the person’s perception of self in individuals with higher levels of FoF; failure to them is an indicator of ineptitude, and it leads them to believe that they are unworthy of love (Elliot and Thrash, 2004). There are five subscales for FoF: fear of shame and embarrassment (FSE), fear of devaluing one’s self-estimate (FDSE), fear of having an uncertain future (FUF), fear of important others losing interest (FIOLI), and fear of upsetting important others (FUIO; Conroy et al., 2002). The Performance Failure Appraisal Inventory (PFAI) instrument was developed by David E. Conroy; it has 25 items that measure belief associated with aversive consequences of failure. It was first validated to measure FoF among young athletes. It was then successfully validated among the Romanian population in educational contexts for the younger population (Holic, 2018).

Fear of failure among students in Saudi Arabia is a relatively under-explored topic. Also, medical students perceive higher stress levels than students in other specialties, but their FoF levels remain unknown (Al-Dabal et al., 2010). FoF must be addressed due to its numerous detrimental effects on medical students, especially in those who are failure avoidant. It is associated with higher anxiety levels, self-doubt, and lower self-estimate (Elliot and Thrash, 2004; Al-Dabal et al., 2010).

The College of Medicine at King Saud University is one of the oldest medical colleges in the region. This college offers a 5-year curriculum in which the first and second years offer basic science, while the third, fourth, and fifth years offer clinical science. The grade point average (GPA) of each year represents the GPA of the year before as GPAs are available after the end of each academic year due to the annual system.

A cross-sectional observational study was conducted. Our objectives were to measure FoF among medical students at King Saud University, to compare FoF between male and female medical students and across their academic levels, and to compare the mean value of FoF across GPA levels and other factors among medical students.

## Materials and Methods

### Participants and Procedure

This cross-sectional study was carried out in the College of Medicine, King Saud University (KSU) in Riyadh. It was conducted during the academic year 2019–2020. The study participants were both male and female undergraduates pursuing medicine in the second, third, fourth, and fifth years. A total of 455 students constituted the study participants who were selected using the stratified random sampling method and were selected through an email, which was sent to their official university email containing the link to an electronic questionnaire designed using google forms.

### Ethical Considerations

Ethical approval was obtained from the Institutional Review Board (IRB) in King Saud University on December 8, 2019, under project number #E-19-4435. Before proceeding with the questionnaire, the purpose of this study and its procedures were explained to all the participants. All the questions of participants were fully answered. All participants understood the explanations and provided online informed consent, and no incentives or reward were given to participants.

### Instruments

The questionnaire consisted of 2 parts, namely, demographic characteristics and the scale to measure FoF, which is the “Performance Failure Appraisal Inventory (PFAI) scale” developed by Conroy, Willow and Metzler (Al-Dabal et al., 2010). The first part of the questionnaire included the demographics, where the participants were asked about 9 questions: gender, academic year (second, third, fourth, and fifth), GPA (<3.5, 3.51–4, 4.01–4.5, 4.5–4.9, and >4.9), how many hours do you study per day, not including school hours? (less than 3 h and more than 3 h), how many hours do you sleep per day? (1–3 h, 4–6 h, and more than 6 h), interest in studying medicine (Did you choose to study medicine because you were interested in the field? Yes/No), siblings in medical school (Do you have any siblings in medical school? Yes/No), previous academic failure during the time of enrollment in the college of medicine (Have you failed a block/subject in medical school? Yes/No), and type of secondary school (public or private schools).

The second part of the questionnaire was the PFAI instrument, and it had 25 items that measured beliefs associated with aversive consequences of failure. Responses to the PFAI are classified based on a five-point scale ranging from −2 to +2 (−2, −1, 0, +1, and +2). The value of −2 indicates “do not believe at all,” 0 indicates “believe 50% of the time,” and +2 indicates “believe 100% of the time.” A score toward “0” and above indicates a higher level of fear. The PFAI measures the strength of individuals’ beliefs in five aversive consequences of failing. Scores are provided for each of these five domains of fears of failing: (a) fear of experiencing shame and embarrassment (FSE), (b) FDSE, (c) FUF, (d) FIOLI, and (e) FUIO. Construct validity evidence has been discovered for this tool (Conroy et al., 2002; Conroy and Metzler, 2003; Holic, 2018).

### Data Collection and Statistical Analysis

Data were collected by our research team in the period between January 29, 2020, and February 25, 2020. Data were analyzed using SPSS 26.0 (IBM Inc., Chicago, United States) version statistical software. Descriptive statistics (mean, SD, median, interquartile range, frequencies, and percentages) were used to describe the quantitative and categorical variables. Bivariate statistical analysis was carried out using appropriate non-parametric statistical tests (the Mann-Whitney *U*-test and the Kruskal-Wallis test followed by the Conover *post hoc* test) to compare the mean rank values of different domains and total score of FoF about the categorical study variables of two and more than two categories. Non-parametric statistical methods were used due to higher SD in domain values and also the statistical significance of the normality test (Ghasemi and Zahediasl, 2012). Internal consistency of the PFAI instrument was assessed using Cronbach’s α (Gliem and Gliem, 2003; Tavakol and Dennick, 2011). Karl Pearson’s correlation coefficient among the items and scores of five domains were calculated to assess the convergent validity. The construct validity of PFAI was determined by using factor analysis in which the correlation matrix, Kaiser-Meyer-Olkin (KMO) measurement of sampling adequacy, and Bartlett’s test of sphericity were used to assess the factorability of the 25 items. By using the principal component method, the factor structure was restricted to five factors in the process of factor extraction. Eigen-values were used to assess the proportion of variance explained by each of the five factors. The rotated factors were obtained by using Varimax rotation (Yong and Pearce, 2013). A *p*-value of ≤0.05 and 95% confidence intervals were used to report the statistical significance and precision of the results.

## Results

Of the 455 study subjects, 227 (49.9%) were men, 117 (25.7%) were from the second year, 121 (26.6%) from the third year, and the remaining 24% and 23.7% were fourth- and fifth-year undergraduate medical students. A higher GPA (>4.9) was held by 24 (5.3%) students, 175 students (38.5%) had a GPA value of 4.5–4.9, and the remaining 256 students (56.3%) had GPA < 4.5. A majority (85.5%) of the study subjects had expressed that they were interested in studying medicine. About 17.1% of them had failed in some of the subjects during their study period and one-third (34.7%) of the subjects had their siblings studying in medical school. More than 60% of them were spending more than 3 h per day as their study hours.

The descriptive statistics of five domains (FSE, FDSE, FUF, FIOLI, and FUIO) and all items of the PFAI are listed in Table 1 The mean values of the 5 domains ranged from −0.0170 to −0.7169 and the median values from −0.500 to 0.000.

**TABLE 1:** Descriptive statistics and reliability—internal consistency (Cronbach’s α) of five domains and fear of failure of the performance failure appraisal inventory.

Name of domains and FoF	Mean (Sd.)	Median (IQR)	Cronbach’s alpha (95% CI)
FSE	−0.0170 (1.022)	0.000 (1.57)	0.860 (0.840, 0.879)
FDSE	−0.3742 (1.001)	−0.500 (1.25)	0.798 (0.766,0.827)
FUF	−0.1891 (0.859)	−0.250 (1.25)	0.575 (0.507,0.635)
FIOLI	−0.7169 (1.035)	−1.000 (1.60)	0.875 (0.856,0.892)
FUIO	−0.2633 (1.056)	−0.400 (1.80)	0.825 (0.798,0.849)
FOF *(All 25 items)*	−0.3117 (0.797)	−0.396 (1.15)	0.931 (0.921,0.940)

*FSE, fear of experiencing shame and embarrassment; FDSE, fear of devaluing one’s self-estimate; FUF, fear of having an uncertain future; FIOLI, fear of important others losing interest; FUIO, fear of upsetting important others; FOF, fear of failure.*

The comparison of each of the 5 FoF domain score mean ranks concerning the characteristics of study subjects shows a statistically significant difference in the mean ranks of FSE, domain scores for GPA, and academic level, where the mean ranks of the subjects who had GPA of <3.5 and >4.9 are statistically significantly higher when compared with the subjects of another level of GPA (*p* = 0.023), also the mean ranks of subjects who were in the third year were significantly higher than the subjects who were in the second, fourth, and fifth years (*p* < 0.0001). This indicates that the FSE scores are significantly higher in these subjects. In addition, mean ranks of FSE domain scores are not statistically different in comparison to the other characteristics of the study subjects. The mean ranks of FDSE domain scores are statistically and significantly higher in female subjects when compared with male subjects (*p* < 0.0001). Also, third-year subjects had a significantly higher FDSE scores when compared with subjects of other academic years (*p* = 0.003). Other characteristics did not show any statistically significant difference toward the mean ranks of the FDSE domain. The mean ranks of FUF domain scores are statistically and significantly higher in subjects who had expressed that they are not interested in studying medicine (*p* = 0.038) and in those subjects who spent less than 3 h studying daily (*p* = 0.011). The mean ranks of FIOLI domain scores are statistically and significantly higher in male subjects (*p* = 0.019) when it is compared with female subjects; significantly higher in subjects who had a GPA of <3.5 and a GPA of >4.9 (*p* = 0.029) when compared with subjects of other GPA levels; and in subjects who had stated that they are not interested in studying medicine (*p* = 0.041) when compared with subjects who are interested in studying medicine. The mean ranks of FUIO domain scores are statistically and significantly higher in subjects who had a GPA of <3.5 and a GPA of >4.9 (*p* = 0.006) when compared with subjects of other GPA levels and third-year subjects having significantly higher FUIO scores when compared with subjects of another academic year (*p* = 0.001). The mean ranks of FoF indicate statistically significant differences concerning GPA, academic level, and interest in studying medicine. That is, the FoF is significantly higher in subjects who had a GPA of <3.5 and a GPA of >4.9 (*p* = 0.012); in subjects who are studying in the third year of their curriculum (*p* = 0.001), and in those who are not interested in studying medicine (*p* = 0.014) (Table 2).

**TABLE 2:** A bivariate analysis using the Mann-Whitney *U* and the Kruskal-Wallis tests to compare the mean ranks of five domains and total score of general FoF and in relation to characteristics of study subjects.

Characteristics	Domains											
	FSE		FDSE		FUF		FIOLI		FUIO		FOF	
	Mean ranks	*p*-value	Mean ranks	*p*-value	Mean ranks	*p*-value	Mean ranks	*p*-value	Mean ranks	*p*-value	Mean ranks	*p*-value
**Gender**												
Male	216.94	0.073	200.10	<0.0001	217.64	0.079	242.43	0.019	235.80	0.206	221.27	0.276
Female	239.01		255.78		239.26		213.64		220.23		234.70	
**GPA**												
<3.5	256.87	0.023	245.60	0.598	255.74	0.150	281.89	0.029	280.55	0.006	277.69	0.012
3.51–4	225.80		227.85		230.64		221.34		236.53		229.35	
4.01–4.5	228.40		223.89		224.73		233.31		216.92		224.24	
4.5–4.9	210.38		219.86		213.86		209.40		210.69		209.53	
>4.9	296.77		259.88		275.63		260.54		288.69		286.27	
**Academic level**												
Second	217.66	<0.0001	218.94	0.003	223.41	0.129	237.95	0.233	224.84	0.001	222.42	0.001
Third	272.89		265.58		252.03		240.48		266.95		268.41	
Fourth	215.52		211.22		221.80		222.47		212.13		212.88	
Fifth	201.50		212.64		214.19		208.82		203.80		204.03	
**Interest in medicine**												
Yes	223.58	0.082	224.48	0.164	223.24	0.038	223.83	0.041	223.56	0.080	221.78	0.014
No	254.04		248.77		259.58		258.50		254.14		264.67	
**Block/year failure**												
Yes	235.78	0.566	224.20	0.778	250.19	0.109	251.31	0.085	253.09	0.064	248.29	0.134
No	226.39		228.79		224.02		223.18		222.81		223.80	
**Siblings in medical** **school**												
Yes	223.99	0.635	227.50	0.953	226.36	0.799	217.33	0.205	218.47	0.259	220.82	0.396
No	230.13		228.27		229.68		233.68		233.07		231.82	
**Study hours in a day**												
<3	232.69	0.560	219.18	0.272	248.97	0.011	232.95	0.538	233.26	0.514	232.86	0.546
>3	225.26		233.16		216.56		225.10		224.92		225.15	

*FSE, fear of experiencing shame and embarrassment; FDSE, fear of devaluing one’s self-estimate; FUF, fear of having an uncertain future; FIOLI, fear of important others losing interest; FUIO, fear of upsetting important others; FOF, fear of failure; GPA, grade point average.*

The internal consistency and reliability of 25 items, five main domains (FSE, FDSE, FUF, FIOLI, and FUIO) were assessed by calculating Cronbach’s α. As we can see in Table 1, the average measure of Cronbach’s α value of all the 25 items is 0.931 and for the five domains range from 0.575 to 0.875. The values of Cronbach’s α are close to the acceptable level of 0.7 for the four domains and overall inventory, which suggests a satisfactory estimate of the reliability of the questionnaire (Gliem and Gliem, 2003; Tavakol and Dennick, 2011; Table 1).

The original validation of the PFAI instrument (Al-Dabal et al., 2010) showed internal consistency values for the five main domains (FSE, FDSE, FUF, FIOLI, and FUIO) as follows: (0.80, 0.74, 0.80, 0.81, and 0.78).

The correlation among the 25 items of an inventory is statistically significant. The KMO measure of the sampling adequacy is 0.935, which is greater than 0.5, thus a satisfactory factor analysis and Bartlett’s tests of sphericity are significant (*p* < 0.0001) (Yong and Pearce, 2013; Gorsuch, 2014), indicating that the correlation matrix is not an identity matrix. The factor extraction along with the Eigenvalues, the percentage of variance attributable to each factor, and the cumulative variance of the factors show that the first factor accounts for 39.71% of the variance, the second factor accounts for 8.85% of the variance, the third factor for 6.53% of the variance, the fourth factor for 5.36% of the variance, and the fifth factor for 4.06% with a cumulative variance of 64.52%. The loadings of the 25 items on the five factors were extracted. The higher the absolute value of the loading, the more the factor contributes to the variable. The loading indicates that five factors have contributed to each of its (7, 4, 4, 5, and 5) items. The five factors are FSE with its seven items (10, 15, 18, 20, 22, 24, and 25), FDSE with its four items (1, 4, 7, and 16), FUF with its four items (2, 5, 8, and 12), FIOLI with its five items (11, 13, 17, 21, and 23), and FUIO with its five items (3, 6, 9, 12, and 19) (Table 3).

**TABLE 3:** Factor loading of five factors of the performance failure appraisal inventory.

The performance failure appraisal inventory		FSE	FDSE	FUF	FIOLI	FUIO
Item no.	Statement					
10	When I am not succeeding, I am less valuable than when I succeed.	0.373				
15	When I am not succeeding, I get down on myself easily.	0.451				
18	When I am failing, it is embarrassing if others are there to see it.	0.657				
20	When I am failing, I believe that everybody knows I am failing.	0.681				
22	When I am failing, I believe that my doubters feel that they were right about me.	0.425				
24	When I am failing, I worry about what others think about me.	0.757				
25	When I am failing, I worry that others may think I am not trying.	0.649				
1	When I am failing, it is often because I am not smart enough to perform successfully.		0.779			
4	When I am failing, I blame my lack of talent.		0.844			
7	When I am failing, I am afraid that I might not have enough talent.		0.835			
16	When I am failing, I hate the fact that I am not in control of the outcome.		0.169			
2	When I am failing, my future seems uncertain.			0.618		
5	When I am failing, I believe that my future plans will change			0.656		
8	When I am failing, it upsets my “plan” for the future.			0.725		
12	When I am failing, I am not worried about it affecting my future plans.			0.617		
11	When I am not succeeding, people are less interested in me.				0.694	
13	When I am not succeeding, people seem to want to help me less.				0.789	
17	When I am not succeeding, people tend to leave me alone.				0.802	
21	When I am not succeeding, some people are not interested in me anymore.				0.745	
23	When I am not succeeding, my value decreases for some people.				0.701	
3	When I am failing, it upsets important others.					0.785
6	When I am failing, I expect to be criticized by important others.					0.441
9	When I am failing, I lose the trust of people who are important to me.					0.365
14	When I am failing, important others are not happy.					0.864
19	When I am failing, important others are disappointed.					0.774

*FSE, fear of experiencing shame and embarrassment; FDSE, fear of devaluing one’s self-estimate; FUF, fear of having an uncertain future; FIOLI, fear of important others losing interest; FUIO, fear of upsetting important others.*

## Discussion

Universally, at all medical schools, the changes in the curricular contents, the teaching methods, and conducting examinations are emphasized greatly. Nevertheless, not much care is given to the students’ capabilities to cope up with the learning and reasons for failure. This study aimed to measure FoF among King Saud University medical students and to assess the reliability and validity of the FoF instrument. The results of our study show that the mean for FoF was −0.3117, indicating that the level of FoF among medical students at King Saud University was low. This finding is similar to the finding of a previous study done among undergraduate students at Hashemite University (Alkhazaleh and Mahasneh, 2016). Low levels of FoF were attributed to the demanding nature of the transition period between high school and university life that forces the students to become more independent and to cope with the social and academic requirements of university life (Alkhazaleh and Mahasneh, 2016). Moreover, we believe that the FoF level was low in our study population due to the personal affective factors of students who pursue a career in medicine (Anvik et al., 2008; Artino et al., 2010).

The results of this study indicate that women had higher levels of FDSE, which supports previous studies that women had higher scores in guilt-proneness and self-criticism (Thompson et al., 2008). However, previous research has also shown that women have higher levels of FSE (Alkhazaleh and Mahasneh, 2016). This was attributed to the educational system that focuses more on the career goals of men than of women (Rothblum, 1990). In our study, the high FIOLI in men might be due to certain social or family expectations regarding the male role in taking responsibilities of the family. In a study done in China about filial obligations and expectations, the results showed that men felt more obliged to assist their family elders financially and more loaded on this aspect than the other familial duties (Yue and Ng, 1999).

The results, as shown in Table 2, indicate that FSE, FDSE, and FUIO are significantly high among third-year subjects when compared with the second, fourth, and fifth-year subjects. A possible explanation for this might be that during the third year, there is a shift from basic science years into the clinical years. This finding supports previous research that found that students at the beginning of their clinical training are afraid of making mistakes and looking awkward in front of patients and senior doctors (Radcliffe and Lester, 2003). Furthermore, there is a variation between different academic characteristics and the level of FoF. Compared with most colleagues, students with a GPA below 3.5 have a high level of FoF, FIOLI, and FUIO. The Stuart investigation has shown supportive results in the correlation between FoF and academic GPA (Stuart, 2013), which leads us to conclude that students exhibiting higher levels of FoF had lower GPA in comparison with their peers with higher academic confidence and lower failure anxiety. One unanticipated finding was that students with a GPA of greater than 4.9 have the highest level of FoF, FIOLI, and FUIO among their peers. However, this result has never been described earlier. This might be due to the association between FoF and procrastination, which was found to be mediated by the level of competence. In students with higher levels of competence, the increase in the level of FoF decreases procrastination; therefore, it increases their academic performance (Haghbin et al., 2012). A study done among third-year medical students at the University of Kansas School of Medicine revealed that students who were afraid of failure had a high proportion of tasks completed successfully (Levant et al., 2020). Findings from this study show that the mean rank of FOF was significantly higher in people who have expressed that they are not interested in medicine. They also had high FUFand FIOLI. Other subjects who were found to have high FUF are students who spent less than 3 h for studying daily.

Students who express course-related enjoyment and interest in studying medicine are more likely to have a higher and more satisfactory academic achievement, deep learning, and well-being compared with those who are not interested that were labeled as amotivated students (Artino et al., 2010; Orsini et al., 2015; Sarkis et al., 2020). Motivation plays a crucial part in any educational system because they influence student conduct as well as their performance and outcome (Kusurkar et al., 2013; Sarkis et al., 2020). Such results indicate that loss of interest and amotivation plays a major role in FoF. Our study results show high reliability with positive levels of internal consistency for the items of five factors and all the items of an FoF instrument. The construct validity of five factors showed a total variance of 64.52%. Similar results of validity and internal consistency of this instrument were reported in multiple studies carried out on Romanian, Malaysian, and Indian populations (Mohanan, 2012; Holic, 2018; Rawat, 2019).

## Limitations

Our study is a single-center cross-sectional study conducted on medical students at King Saud University; therefore, it may be difficult to generalize the results. Our questionnaire is also an online self-administered questionnaire and not interview-based; therefore, the reliability of the results could be questioned. In addition, the reliability of one of the five factors, FUF, is 0.575, which is lower than the acceptable level of 0.7. This could be due to the lack of understanding of the five items under this factor. But the overall reliability of the instrument, which consists of 25 items, is 0.931, which is very good and greater than the acceptable level. The question addressing sleeping hours per day in the demographic characteristics section in the questionnaire was a general question with no specification of the period. This was done to avoid the inaccuracy that might happen if a period was specified as it could be an exam period among some of the years and exam-free among others. However, general questions could carry different interpretations depending on the reader.

## Conclusion

The results of this study show that the level of FoF among medical students at King Saud University was low; moreover, the study found higher levels of FDSE in women than in men, and higher levels of FIOLI in men compared with that in women. Also, third-year academic level students anticipated higher FSE and FUIO and FoF compared with others. High levels of FoF were seen in those who have a GPA below 3.5 and a GPA greater than 4.9. Students who were not interested in studying medicine showed a higher level of FoF. More effort should be directed toward creating a smoother transition for medical students between basic science years and clinical years, to decrease the high FoF levels that were seen in third-year students. Also, students who have a GPA below 3.5 and a GPA greater than 4.9 should be advised to visit the student counselor as they have a higher risk of having high FoF. Furthermore, this study confirms the internal consistency and constructs validity of the FoF instrument.

## Data Availability Statement

The raw data supporting the conclusions of this article will be made available by the authors, without undue reservation.

## Ethics Statement

The studies involving human participants were reviewed and approved by the Institutional Review Board (IRB) in King Saud University on the 8th of December 2019 under project number #E-19-4435. The patients/participants provided their written informed consent to participate in this study.

## Author Contributions

All authors conceptualized the study, organized the database, contributed to the study design, collected, analyzed, and interpreted data, and wrote the manuscript.

## Conflict of Interest

The authors declare that the research was conducted in the absence of any commercial or financial relationships that could be construed as a potential conflict of interest.

## Publisher’s Note

All claims expressed in this article are solely those of the authors and do not necessarily represent those of their affiliated organizations, or those of the publisher, the editors and the reviewers. Any product that may be evaluated in this article, or claim that may be made by its manufacturer, is not guaranteed or endorsed by the publisher.
